# Supplementary material for: DMRforPairs: identifying Differentially Methylated Regions between unique samples using array based methylation profiles
Source: BMC Bioinformatics. 2014 May 15;15:141. doi: 10.1186/1471-2105-15-141 (PMC4046028; doi:10.1186/1471-2105-15-141)
Supplement: Additional file 2 — DMRforPairs output for the comparison of A431-MCF7 and NA17018-NA17105. Please start from the HTML files in each folder. Available via the BMC Bioinformatics website. [file 1471-2105-15-141-S2.zip › 1394847754114233_MOESM2_ESM/A431_MCF7/figures/27898.pdf]

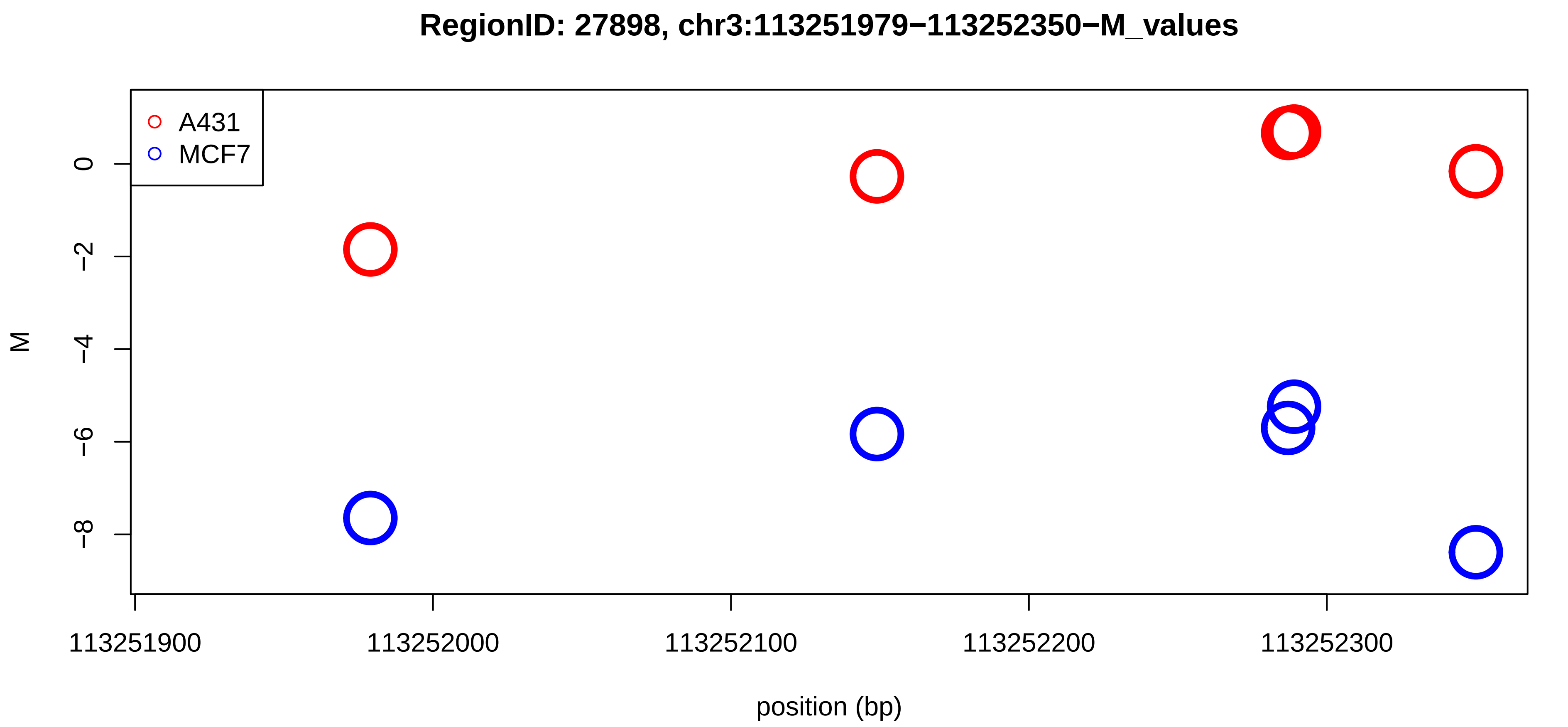

RegionID: 27898, chr3:113251979−113252350−M_values
A431
MCF7
M
0
−2
−4
−6
−8
113251900
113252000
113252100
113252200
113252300
position (bp)


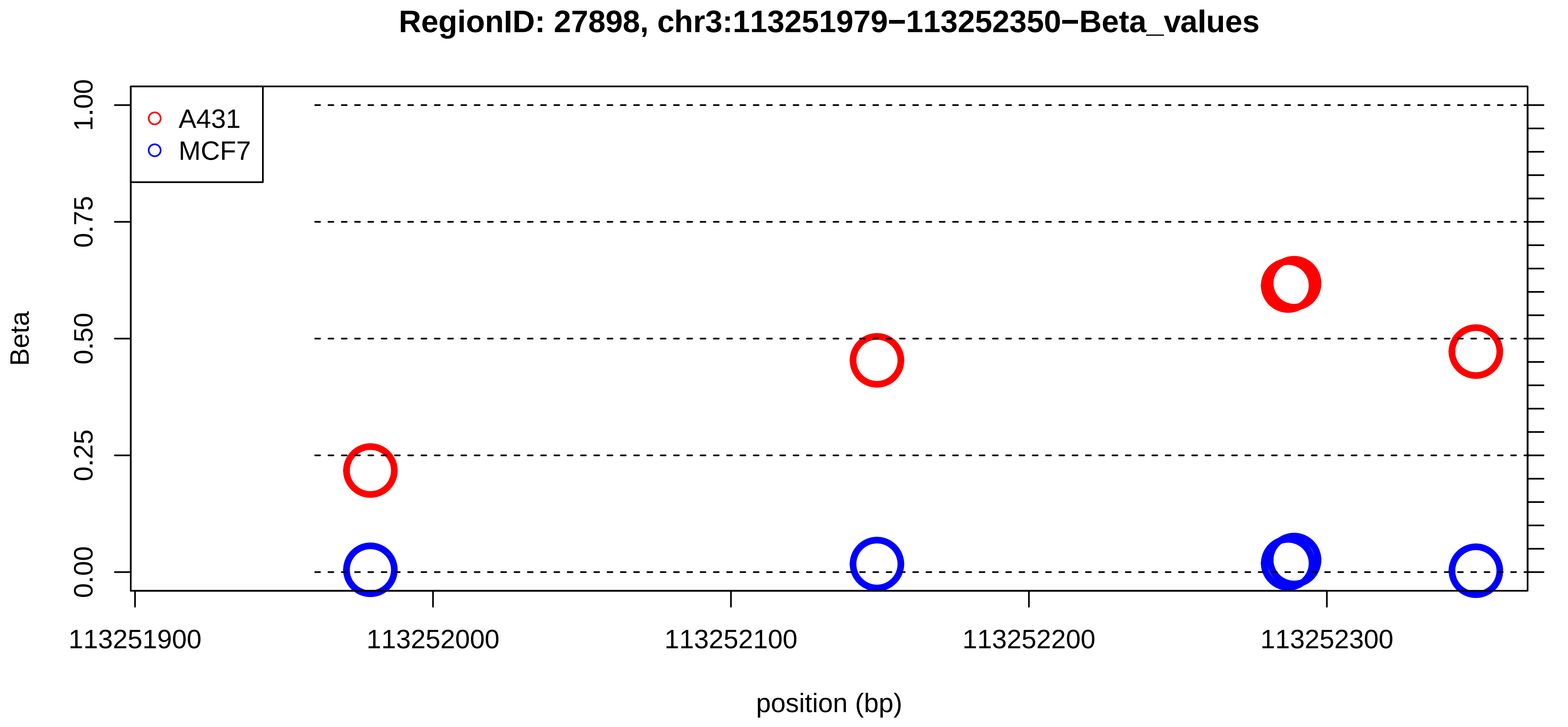

RegionID: 27898, chr3:113251979−113252350−Beta_values
A431
MCF7
Beta
1.00
0.75
0.50
0.25
0.00
113251900
113252000
113252100
113252200
113252300
position (bp)

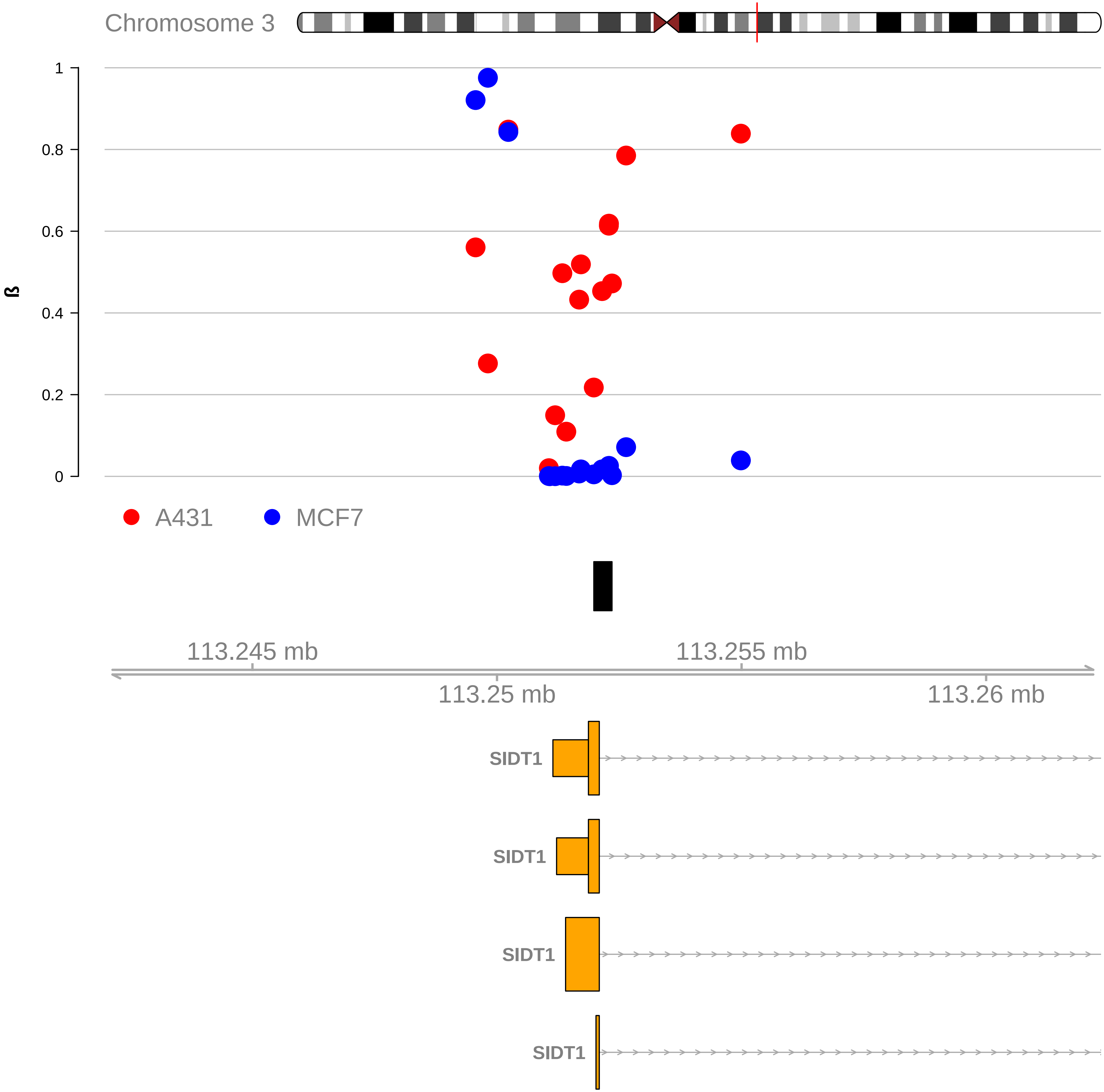

Chromosome 3
ß
1
0.8
0.6
0.4
0.2
0
A431
MCF7
113.245 mb
113.255 mb
113.25 mb
113.26 mb
SIDT1
SIDT1
SIDT1
SIDT1
